# Reports on Hospitals of the United Kingdom

**Published:** 1913-12-06

**Authors:** Henry Burdett


### REPORTS ON

### Hospitals of the United Kingdom.

By SIR HENRY BURDETT, K.C.B., K.C.V.O.

SERIES III.

### THE EAST SUFFOLK AND IPSWICH HOSPITAL, IPSWICH.

This institution was established in 1836. In 1869 a storey was added to the original structure and wards for women were built out to the west and for men to the east, in two-storeyed pavilions. Eight years later the women's pavilion was extended at the end to provide accommodation for children, and ten years afterwards the men's accommodation was increased by the erection of two wards in a pavilion running north and south. In 1891 the chapel was built to the north-east, and the next year saw the addition of the Nurses' Home with cubicles instead of separate rooms, but on the enlargement of the home in 1904 this mistake was not repeated in the new buildings. In the latter year the new Bartlet Out-patient Department was erected. It will thus be seen that the needs of the inhabitants of Ipswich for in-patient treatment began to be apprehended, and that for the twenty years that followed, the committee, with the co-operation of some generous givers, laboured to bring the buildings to a high state of efficiency. It may be useful to remark here, as has been appreciated in connection with some large hospitals in London, that it would have been better and more economical, in the administrative sense especially, to have had plans prepared in 1877 for the rebuilding of the whole hospital, so as to make the best possible use of the site and to secure the ready administration and working of the hospital when completed. He would have been a bold man with a great mind and no little courage who would have brought forward in 1877 and pressed for adoption a great building scheme, such as that just referred to. In its absence, by 1907, the committee and people of Ipswich woke up to the fact that many structural improvements were still called for, and it was then determined to undertake an important building scheme, involving a total expenditure of some £20,000. In the three years which followed, a new administration block to the north of the central block, a new operation theatre, and complete unit were erected, together with a detached isolation block in the north-west corner of the grounds, and an additional range of buildings, comprising a laundry, mortuary, and other structures, was placed on the north-east border of the hospital site. In addition to this, material internal changes were made in the old buildings with the object of modernising them as much as possible.

The mere mention of the extensive reconstructions and new buildings which have been added to this hospital during the last twenty-five years testifies to the progressive spirit of the governing authorities, and reflects infinite credit upon everybody who has had a hand in the work. Ipswich, of course, has not obtained a thoroughly new and up-to-date hospital, but it has at the present time a mass of buildings which collectively provide for the treatment of patients upon modern lines, though the work has to be carried out at a greater cost, than would have been the case, had it been possible to anticipate the extent of the work the hospital has to do at the present day. Such foresight would allow provision to be made for the future from the moment when the necessity for additional buildings and a scheme of reconstruction first became apparent.

### Some Openings for Wise Capitalists.

We hope that in the course of 1914 the first portion of the Nurses' Home will be reconstructed, so as to do away with the existing cubicles and provide a separate room for each nurse at present housed in these portions of the buildings. We have to congratulate the committee upon the considerable addition to the means of treatment afforded by the Pamela Cobbold Balconies to the children's ward, and we hope that balconies will soon be added to the main wards, on a plan of construction that will not cause them to interfere in any way with the light and air of any portion of those wards, whilst they will be wide enough to allow the patients' beds to be placed at right angles to the building, and so to accommodate the majority of the patients in each ward if or when it is deemed necessary.

A fine addition has been made to the out-patients' department by the new electric baths and massage sections, together with an operation theatre and other modern adjuncts for treatment to be found in the new annexe. The plan, scope, and ample space afforded in this annexe constitute it one of the most attractive features of the East Suffolk and Ipswich Hospital to-day. This section also provides preparation and recovery rooms, a waiting room, and a well-planned casualty room, divided into two departments, one for men and one for women.

### Some Results of Inspection.

Our inspection of the hospital on October 20, 1913, affirmed the general criticisms we have already given. The wards are pleasant, airy apartments, with central back-to-back slow-combustion grates—a type of fireplace fitted throughout the new buildings—which cannot, we believe, give sufficient heat to warm thoroughly an entire ward in the severer season of the year. Many of the older portions of the hospital have been refloored, and the old matchboarding dadoes have been replaced by white tiles, with a grey-blue glazed moulding at the top. All the corridors are laid with linoleum. The men's surgical wards contain fifteen and eleven beds respectively, constituting a ward unit of twenty-six beds, in charge of a sister. The unit is made up of two small wards and a duty room, rooms for linen and patients' clothes, with bath and sink rooms situated in the middle of the Goodrich and Victoria wards, where much space is taken up by a section which at first sight resembles a tomb in St. Peter's, Rome, but on examination turns out to be an enclosed staircase which has passed out of use. The wards are lighted by single pendants down the centre and, in addition, there are electric lamps hanging on the walls between each pair of beds. These latter lights are available when required, and in case only one patient desires a light the standard is placed on the locker nearest to the reader in a way which shuts off the light from the other bed. The Mary women's ward is a counterpart of the Goodrich ward, just described, except that it contains sixteen beds, but in place of the Victoria ward the Cobbold children's ward has been erected with the very excellent balconies already alluded to, which are so placed as to render it possible to carry out open-air treatment and to form a playground for the children in practically all weathers.

### The Operation Theatre and Rontgen Rays.

The operation theatre is placed in an entirely new building and is fully large according to modern notions. There is an anæsthetising room and a sterilising room which communicates with the theatre by an open archway; its appliances are similar to those in use at Edinburgh and at the Royal Infirmary, Newcastle. The anæsthetising room is fitted with an outside steel revolving shutter to enable it to be used as a theatre for ophthalmic operations performed by artificial light. The walls of the entire annexe are covered with white tiles and the floors are "terrazzo." The heating is effected by large hot-water radiators with louvre inlets behind them for ventilating purposes. Exhaust fans are used to facilitate the rapid exchange of the air when desired. The Röntgen ray department is fitted with all kinds of apparatus, together with a small motor transformer used for electro-therapy.

### The Domestic Departments.

The kitchens and scullery are of ample size, well lighted and ventilated, the cooking being done partly by steam and partly by gas. The food is distributed in hot-water jacketed containers, one for each ward, which are planned to contain separate rectangular tin boxes with covers and divisions for the food as ordered in the diet sheets. A separate tin box is provided for meat, poultry, and potatoes, another for fish, another for green vegetables, and a fourth for puddings. The containers, when ready for removal to the wards, are packed in special wagons, running on rubber-tyred wheels. On the whole we consider this arrangement for food distribution to offer many advantages, and it is claimed, and we can fully believe, that it provides that all food reaches the patients in a hot and appetising form. There is a dry and extensive basement in which stores are kept, with a good larder, tiled throughout, a developing room for photographic plates, boilers, and storage cylinders for hot-water supply, and also the hot-water boiler to serve the radiators in the north and central portions of the hospital.

The out-patients' department contains a central waiting-hall, with consulting and examination rooms, together with a surgery, lavatories, dispensary, and refreshment buffet. This building is heated by hot water throughout.

### Some Minor Departments.

The isolation block consists of two floors and contains four wards, each of which is capable of receiving four patients, and provides for the upper floor to be entirely isolated from the lower when desired. Each floor has a bathroom, a kitchen, with cooking-range and hot-water boiler, together with larders for food. In the attics of this block three bedrooms are placed for the accommodation of the nurses.

The reconstruction has provided this hospital with ample means for dealing effectually with infected material, the cleansing of mattresses, and general sterilisation. The arrangement of the steam-jacketed sterilisers is good, as it permits infected mattresses, clothes, etc., to be kept wholly apart and separate, so that it is possible to place these articles in the disinfector at one side and to take them out cleansed at the other and ready for use. There is also an ample laundry.

We are glad to note that this hospital is provided with an excellent bacteriological laboratory, together with a complete pathological section and an up-to-date mortuary.

### Faulty Administration Needing Reform.

Taking all together the reconstruction of this hospital has been carried out with commendable enterprise and thoroughness. We were glad to learn that the staff contains many active workers with considerable scientific ability, and that, so far as the internal and scientific sides of the work are concerned, the maximum of efficiency is maintained. We were sorry to hear, however, that too little consideration is shown for the out-patients, owing to the irregular attendance and want of punctuality of the staff attached to this department. The committee must be fully conscious that time is of the first importance to the industrial members of the population of a great town like Ipswich, and that the regulations ought to be so drawn as to ensure that every member of the medical staff shall be in punctual attendance. Failing this, that continued irregularity and consequent loss to patients should be prevented by a rule, which entails the resignation of any member of the staff who fails to be habitually in attendance at the hour when the work of the out-patient department is advertised to begin. We gather from a study of the report and the way in which the names of the medical staff are given on page 7 that, in fact, strange as it may appear, this hospital has no physicians and no surgeons, as such, upon its honorary staff. Has not the time come in the best interests of the medical profession, the hospital, and the patients for the appointment of a committee, to whom the present regulations and medical arrangements of the hospital should be referred with a view to reorganisation and improvements upon modern up-to-date lines? It would appear from the report that each member of the medical staff acts as a physician or as a surgeon, as the circumstances of each case may require. This dual capacity of the members of the medical staff seems to have prevented the adoption of regulations for the admission of patients which are essential nowadays to the proper conduct of a modern hospital. It would appear that no member of the medical staff has an allotted number of medical or surgical beds for which he is responsible, but that each member takes his patients as they come, whether they be medical or surgical cases, the probability being that the latter constitute a majority. Another point of importance is that the medical staff does not contain the name of even one anæsthetist, whereas there should be a number of qualified anæsthetists attached to this hospital, having regard to the number of operations performed under general anæsthetics, which amounted to 1,763 for the year ending May 31, 1913. We believe, too, that this is the only large provincial hospital left which has not yet established a special department for the ear, nose, and throat. These, and other matters relating to the medical service, and the appointment and work of the honorary medical staff, should receive immediate attention with a view to bringing all such matters into line with modern practice governing hospitals of 100 beds and upwards.

It was a great pleasure to spend some hours in this hospital and to study the conscientious efforts made by those responsible for the reconstruction, refitting, and the addition of up-to-date appliances to bring every department up to date. In the matron's department, as well as in the medical and surgical departments and generally, we found gratifying evidence of a desire to leave nothing undone to maintain the efficiency and promote the comfort and welfare of the patients. We were greatly indebted to the matron and secretary for their courtesy and knowledge. We have already expressed our approbation of the ability displayed in the production of the annual report (The Hospital, October 18, 1913, page 60).

### Finance.

An examination of the income and expenditure over a series of years shows that the main source of income, including subscriptions, workmen's and congregational collections, and donations, is well maintained. The average yield is good, which reveals an encouraging tendency in growth during the three years ended May 1913 especially. Despite the large expenditure entailed by the thorough reconstruction of the hospital the amount of invested property has been well maintained. All this is encouraging, but the present high efficiency of the whole hospital and the character of the work done, have necessarily increased the number of patients and the annual cost of maintenance and treatment. We have recently published some articles on hospital statistics, and we may express the opinion, having regard to the fulness of the statistical tables which relate to the business side of this hospital's work, that the medical statistics should be fuller in future annual reports than they are at present, and that they should include a table giving, in comparative form, the number of operations, with the number of "in" and "out" patients and out-patient attendances for the previous seven years. Two years is not a long enough period to show the development and actual increase in the work in an effective manner and to prove convincingly the causes of the increased cost of the work.

### The Responsibility of Ipswich's Citizens.

Ipswich has an up-to-date hospital, but the inhabitants of Ipswich apparently have not yet realised that this gratifying fact imposes upon every man and woman resident in the area which the hospital serves a much increased responsibility. It consequence of this the hospital at Ipswich, like many hospitals elsewhere, has an urgent need at the moment, *i.e.*, a recognition on the part of the committee and, through them, of the population throughout Ipswich and the district, that from £1,500 to £2,000 a year additional income should be promptly provided. From the business point of view the justice of this claim will surely be recognised in an industrial centre of the importance of Ipswich, when we point out that the in-patients are not hurriedly discharged, but that they are retained until their physical condition is such as to warrant their returning home with a view to their resuming work as breadwinners. This important factor in a modern hospital's work would be materially advanced by the establishment of a semi-convalescent home in connection with the hospital, similar to that connected with the Leeds Infirmary and other provincial hospitals, where patients suffering from open wounds and needing dressings and nursing in the purest air can be removed to complete their cure, and to ensure that, on their discharge, they should be perfectly capable of resuming work with full earning power. What Leeds has done Ipswich can certainly do, for it has become the envy of many smaller towns in the United Kingdom, owing to the prosperity of its industrial establishments and the fame rightly attaching to many of the firms that produce things of high capacity which command the market. This semi-convalescent home might be commenced on an approved and carefully thought out plan, which would enable it to feel its way by not overbuilding at the outset, and yet be capable of expansion, so as to enforce the utmost economy and to provide the greatest facilities for easy working.

When we add to the original cost of maintenance that of the semi-convalescent branch we find that this hospital needs an extension of income of at least £3,000 a year. The present high state of efficiency of the various departments throughout the hospital, as a competent visitor can see for himself, and the relatively small cost at which the work is now done, should stimulate every thoughtful inhabitant to help to provide, with the shortest possible delay, this extra income of £3,000 a year. It is a glorious act for any healthy man or woman deliberately to set apart some portion of each year for personal service in raising funds for the hospital in their midst. We would suggest that a good plan would be to take the wards of the city and to appoint a president of each ward, whose duty it would be to find and select an adequate number of good people willing to give their time and to become captains of streets. The privilege and duty of each captain would be to give the necessary time for interviewing every householder in his or her street and to try to induce them to agree to give at least 1s. or upwards a year, in monthly contributions of 1d. or upwards, which contributions would be called for each month on a day most convenient to the givers. Every monthly subscriber should have the title of associate, and every president, captain of a street, and associate should have a certificate signed by the Mayor and countersigned by the ward president of his district, so that each worker and subscriber might have it framed and hung up in his own particular room or sanctum, as a reminder and stimulus to persevere with the work as an act of praise and thankfulness to God for the blessings of health.

Ipswich has a population of nearly 80,000, so that it must contain in all probability something like 16,000 families or householders. Now, if 10,000 of these contributed 1s. a year, 4,000 of the remainder, on an average, gave 2s. 6d. a year, 1,000, on an average, 5s., 500 an average of 10s., and the remaining 500 gave £1 each during the year, the total would amount to £2,000, *i.e.* two-thirds of the £3,000 of increased income needed. The last £1,000 should be readily forthcoming from the present subscribers, who might collectively be asked to raise their contributions so as to produce this amount, and so complete the £3,000 a year of extra income which is required to defray the cost of administering this hospital efficiently.

Suggested Semi-Convalescent Home.

The income for the semi-convalescent home is a matter which not only affects the patients who are workers with families, but also their employers, and we would venture to suggest that the working men and the employers of Ipswich and district might, in view of what is done elsewhere, properly and readily contribute the income amongst them for the maintenance of the semi-convalescent branch, and so perfect and complete the work of this great institution. The adoption of some such plan of profit-sharing in health and prosperity, throughout the town of Ipswich, would free the community from periodical and regular appeals for funds for the hospital, and would constitute something which not only Ipswich but the county of Suffolk might well be proud of.

